# Analysis of mechanisms underlying accelerated plant growth induced by *NtGLK85* overexpression in tobacco

**DOI:** 10.1038/s41598-025-24323-x

**Published:** 2025-11-18

**Authors:** Xuansong Yang, Hong Chen, Chengliang Chen, Gang Gu, Binghui Zhang, Fei Wang, Dongwang He, Wencan Chen, Zhenping Huang, Zhiping Zhong, Xiaofang Xie

**Affiliations:** 1 College of Life Sciences, Fujian Agriculture & Forestry University, Fuzhou, 350002 China; 2 Fujian Key Laboratory of Crop Breeding by Design, Fujian Agriculture & Forestry University, Fuzhou, 350002 China; 3 Jianning Branch of Sanming Tobacco Company, Sanming, 354500 China; 4 Institute of Tobacco Science, Fujian Provincial Tobacco Company, Fuzhou, 350003 China; 5 Changting Branch of Longyan Tobacco Company, Longyan, 364000 China

**Keywords:** NtGLK85, Tobacco (*Nicotiana tabacum* L.), Photosynthesis, Chlorophyll, Carbon assimilation, Transcriptome analysis, Genetics, Plant sciences

## Abstract

The Golden2-like (*GLK)* genes play a crucial role in chloroplast development in angiosperms. Our previous study revealed a strong correlation between the expression level of the *NtGLK85* gene and the development and maturation of tobacco leaves. Therefore, exploring the regulatory mechanisms controlled by *NtGLK85* during plant growth and development is of significant importance. We generated an overexpression line of *NtGLK85* in the tobacco cultivar K326, designated as D710. Both the transgenic lines (T2) and its control K326 were planted in the field. Phenotypic investigation found that D710 exhibited a faster growth rate and shorter growth cycle compared to the wild-type K326. Samples were collected at three stages: 30 days (A), 45 days (B), and 65 days (C) after transplantation. Physiological and biochemical analysis showed that, at stages A and B, D710 had higher chlorophyll content, total sugar, starch levels, and plant height compared to K326. However, at stage C, the chlorophyll content, total sugar, and starch levels in D710 were lower than in K326, although the plant height was similar. Transcriptome sequencing (RNA-Seq) was performed on samples from all three stages, generating a total of 374,627,262 clean reads. Comparative analysis of the data between K326 and D710 at the three stages identified 1,663 differentially expressed genes (DEGs) at stage A, 1,320 DEGs at stage B, and 1,244 DEGs at stage C. GO and KEGG analysis revealed that these DEGs were predominated associated with plant growth and development. Significant differences were observed in the expression of genes involved in five key pathways related to plant growth and development, including photosynthesis (map00195), fatty acid biosynthesis (map00061), cutin, suberin and wax biosynthesis (map00073), carbon fixation in photosynthetic organisms (map00710), and fatty acid elongation (map00062). These differences were attributed to the *NtGLK85* overexpression. The overexpression of *NtGLK85* increases chlorophyll content in the tobacco leaves, thereby enhancing carbohydrate synthesis and accumulation during the early growth stages. As a result, the D710 line exhibited accelerated growth and a shortened life cycle compared to the wild-type K326.

**Supplementary Information:**

The online version contains supplementary material available at 10.1038/s41598-025-24323-x.

## Introduction

Photosynthesis is a critical process that fuels life on Earth by converting light energy into chemical energy. The development of photosynthetic capacity relies on the close cooperation between organelles, involving tasks such as regulating light perception, gene expression, and the production of essential compounds. It has been reported that introducing novel genetic diversity may enhance photosynthesis in crop plants^1^. Chloroplasts are the crucial organelles for photosynthetic eukaryotes and are essential for all plant life^2,3^. The signaling role of chloroplasts extends beyond photosynthesis to include the transmission of systemic signals and the synthesis of phytohormones, which regulate various aspects of plant development^4^. Recent studies have indicated that chloroplasts have evolved from symbiotic relationships with cyanobacteria^5,6^. Thus, the coordination of photosynthetic organs assembly relies on the synergy between the nucleus and chloroplast. Proplastids in subepidermal meristem cells can transform into mesophyll chloroplasts under light^7^. Additionally, plastids are involved not only in photosynthesis but also in synthesizing important compounds such as amino acids, fatty acids, and various pigments^8–10^.

The Golden 2-like (GLK) transcription factor belongs to the GARP superfamily of nuclear transcription factors^11^, which was first identified in maize (*Zea mays* L.)^12^. It has been found that *GLK* genes are essential in angiosperm chloroplast development, and members of the GLK family play a crucial role in regulating the appearance of chloroplasts during the transition and maturity stages^3,13^. Similarly, *SlGLK2* influences photosynthesis during fruit development and contributes to the characteristics of mature fruits in tomato (*Solanum lycopersicum* L.)^14^. In *Arabidopsis*, both *AtGLK1* and *AtGLK2* genes have been found to be involved in the production of chloroplast redundantly^15,16^. *ZmGLK1* is considered as a regulator of chloroplast development in mesophyll cells of C4 tissues, while in C3 species, *GLK* gene pairs exhibite redundancy and promote chloroplasts development in maize^17,18^. Knocking out *GLK* genes in Arabidopsis^15^, rice^19^, tomato^20^, and moss^16^ has suggested that *GLK1* serves as a downstream regulator, playing a significant role in regulating leaf senescence and leading to a substantial reduction in chlorophyll levels within photosynthetic tissues. Conversely, the overexpression of *GLK* genes has been shown to result in chlorophyll accumulation in non-photosynthetic tissues, as evidenced by studies in *Arabidopsis* roots^21^, rice callus tissues^22^, and tomato fruits^23^. Furthermore, the *GLK* genes can promote a series of nuclear photosynthetic genes to adapt to the environmental and developmental variations by optimizing the photosynthetic ability. Chloroplasts are the major components for carbon fixation and sugar manufacturing within plant cells^24^, while carbohydrate production is strongly correlated with the floral initiation. Therefore, overexpression of *GLK* genes will promote sugar accumulation in plants and thereby promote growth and development. For example, the overexpression of *SlGLK2* enhanced the expression of chloroplast development and fruit photosynthesis-related genes, leading to increased levels of carbohydrates and carotenoids in ripe fruit^20^. In addition, *GLK* gene serves multiple functions in plant growth and development. It regulates plant nitrogen-phosphorus metabolism^25^, controls stomatal opening and closure by modulating the activity of *GLK1/2*^26^, acts as a negative regulator in ABA response^27^, participates in various disease defense responses^18,28,29^, regulates light-induced transcriptional network^30^, and influences leaf senescence^31^.

Tobacco (*Nicotiana tabacum* L.) serves as an important model organism in plant biology. Leaves are the primary organs of photosynthesis in plants, pivotal for the synthesis and accumulation of plant nutrients. During the phase of leaf senescence, a meticulously orchestrated series of structural, metabolic, and genetic transformations occur, notably including the depletion of chlorophyll content, and diminishment of photosynthetic capacity. Considering that leaves serve as the primary harvesting organ of tobacco, investigating the dynamics of plant growth, development, senescence, and internal material transport in tobacco holds particular significance for advancing our understanding of plant physiology and development^32^. The GLK transcription factors play a positive role in transcriptional regulation affecting the expression of photosynthesis-related genes and are important in regulating chloroplast formation, development, and senescence in plants^33,34^. Thus, elucidating the functional roles of members is of great significance. In our previous study, we conducted a comprehensive investigation of the *GLK* gene family members using current tobacco genome sequence data. We observed a decreasing trend in the expression level of *NtGLK85* (*Nitab4.5_0010689g0010*) with increasing senescence levels^35^, suggesting its potential importance in tobacco leaf development and senescence. In this study, we constructed an overexpression material of *NtGLK85*. Agronomic traits assessment of transgenic plants and biochemical substances analysis revealed that overexpressing *NtGLK85* promotes sugar accumulation in plants, thereby promoting growth and development and consequently shortening the plant’s life cycle. Consequently, we integrated biochemical substances and transcriptome profiling to explore the regulatory mechanisms controlled by *NtGLK85* during plant growth.

## Results

### Changes in phenotypic characteristics of *NtGLK85* overexpression materials

**Fig. 1:**
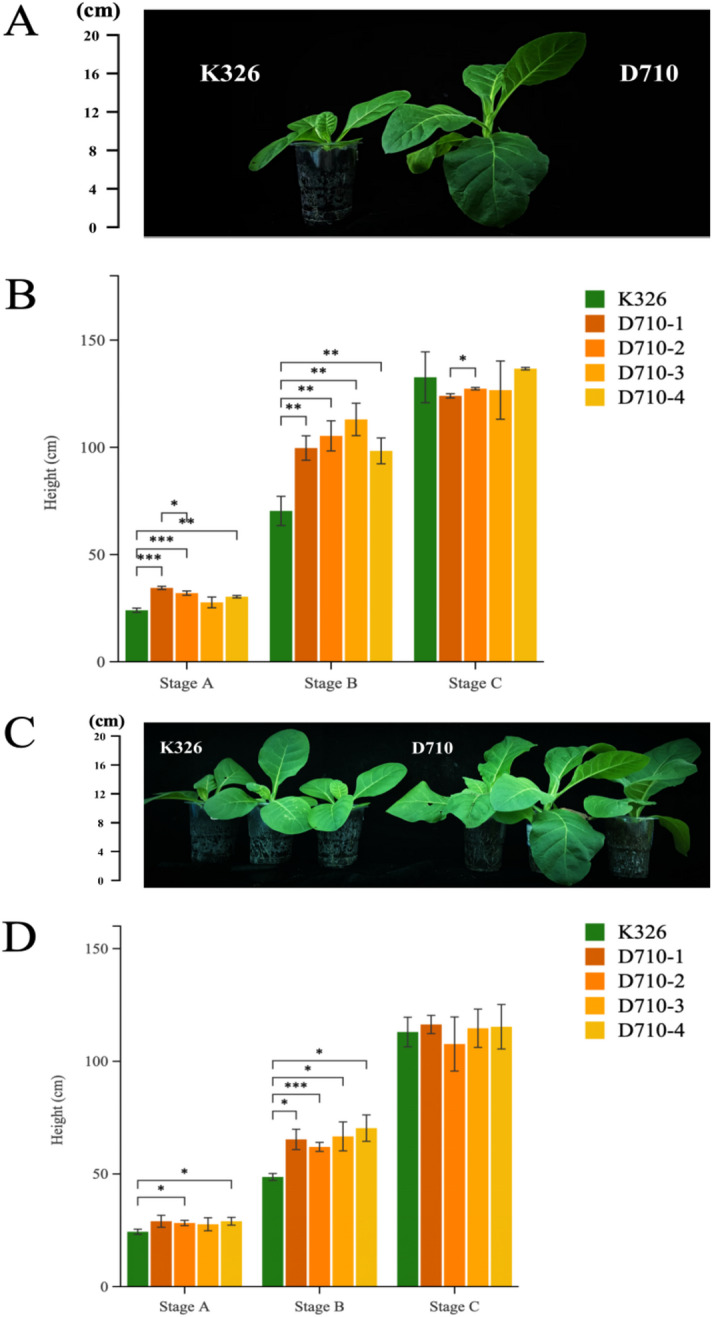
Difference in plant height between K326 and D710: (**A**) Phenotypic differences between K326 and D710 plants in the laboratory in 2022 (**B**) Plant height of K326 and D710 at different growth stages in 2022; (**C**) Phenotypic differences between K326 and D710 plants in the laboratory in 2023; (**D**) Plant height of K326 and D710 at different growth stages in 2023. Stage A: 30 days post-transplantation, Stage B: 45 days post-transplantation, Stage C: 65 days post-transplantation. Error bars indicated the means ± SD (*n* = 30); Statistical significance was assessed using Student’s t-test, *P* ≤ 0.05 as *, *P* ≤ 0.01 as **, and *P* ≤ 0.001 as ***.

**Fig. 2:**
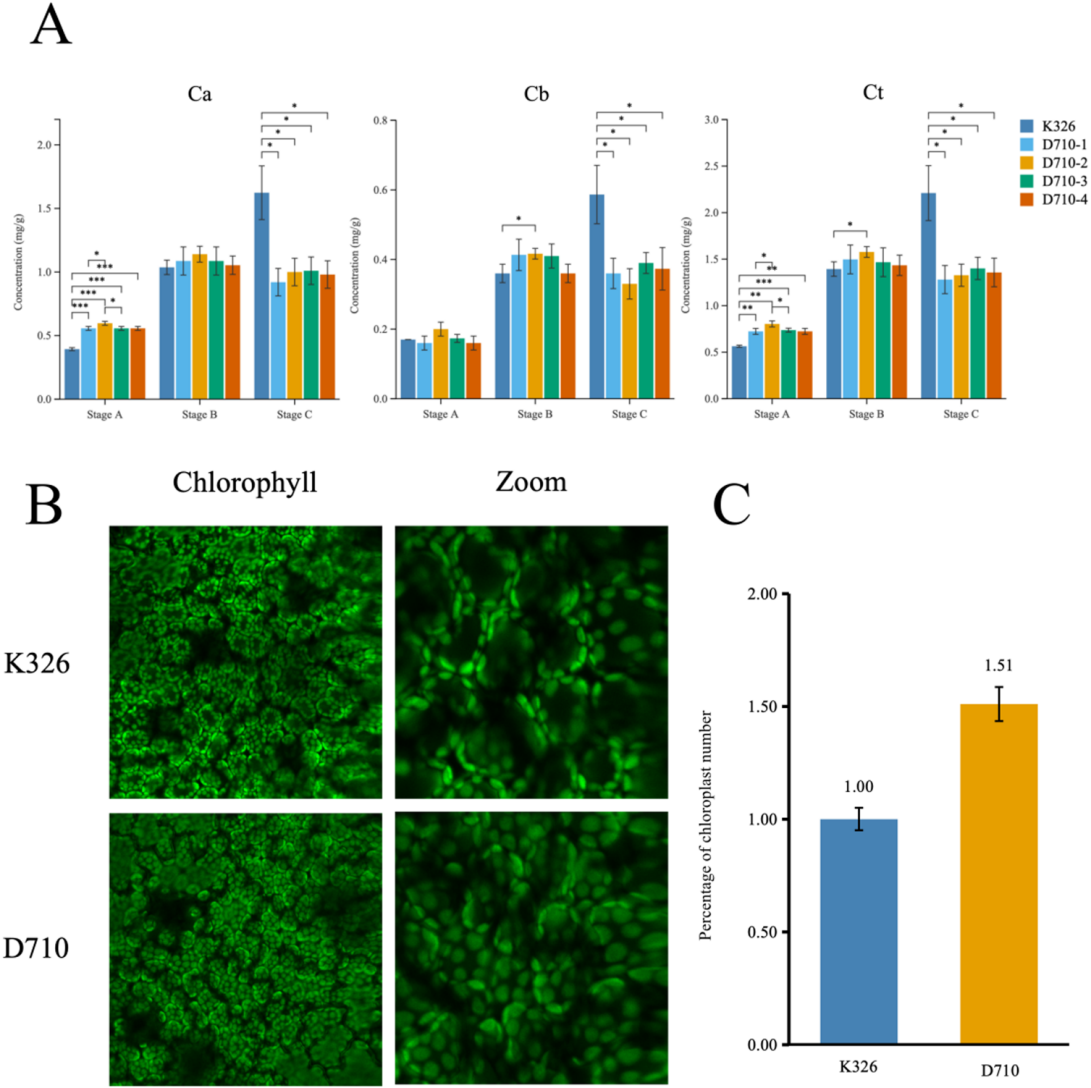
Changes in chloroplast and chlorophyll: (**A**) Chlorophyll a, b, and total chlorophyll content of K326 and D710-1/2/3/4 at different stages (**B**) Images of chloroplast numbers in new leaves of K326 and D710 at stage A under confocal microscope; (**C**) Percentage of chloroplast numbers in new leaves at stage A.

A total of 4 independent *NtGLK85* transgenic plants (D710-1/2/3/4) were obtained from K326. Phenotypic investigations revealed height differences between cultivars K326 and D710 (Fig. 1A and C) under laboratory conditions. To validate this trait, we transplanted these four independent lines to the field in 2022 and 2023. Initial observations showed no height difference at the seedling stage, but a noticeable disparity emerging 30 days after transplantation (Fig. 1B and D, Stage A) in both years, with D710 exhibiting a faster growth rate than K326. At 45 days post-transplantation (Fig. 1B and D, Stage B), D710 plants had significantly surpassed K326 in height. At 65 days post-transplantation (Figs. 1D and 2B, Stage C), the plant heights of both cultivars were similar. Additionally, budding began in D710 at this stage, whereas K326 did not exhibit this phenomenon.

To further compare the growth differences between K326 and D710, we collected middle leaf samples at three developmental stages (A, B, and C) and analyzed their chlorophyll a content. The results revealed that at stage A, the chlorophyll a content in D710-1/2/3/4 was significantly higher than that in K326. While the chlorophyll b content in D710-2 was higher than in K326, the difference was not statistically significant. In contrast, the total chlorophyll content in D710-1, D710-2, and D710-4 was significantly higher, and in D710-3, it was extremely significantly higher compared to K326. At stage B, the contents of chlorophyll a, chlorophyll b, and total chlorophyll in all four transgenic lines (D710-1/2/3/4) were higher than those in K326, with chlorophyll b and total chlorophyll in D710-2 showing statistically significant differences. At stage C, all chlorophyll contents in D710-1/2/3/4 were significantly lower than those in K326, with statistically significant differences (Fig. 2). To further investigate changes in chlorophyll content in transgenic plants, newly developed leaves at stage A were collected for sectioning and analyzed using confocal microscopy. The analysis confirmed that the transgenic plants exhibited a significantly higher density of chloroplasts per unit area compared to K326 (Fig. 2B and C).

Stage A: 30 days post-transplantation, Stage B: 45 days post-transplantation, Stage C: 65 days post-transplantation. Error bars represent the means ± SD (*n* = 30); Statistical significance was assessed using Student’s t-test, with significance levels denoted as follows: * for *P* ≤ 0.05, ** for *P* ≤ 0.01, and *** for *P* ≤ 0.001.

### Verification of *NtGLK85* overexpression levels

**Fig. 3:**
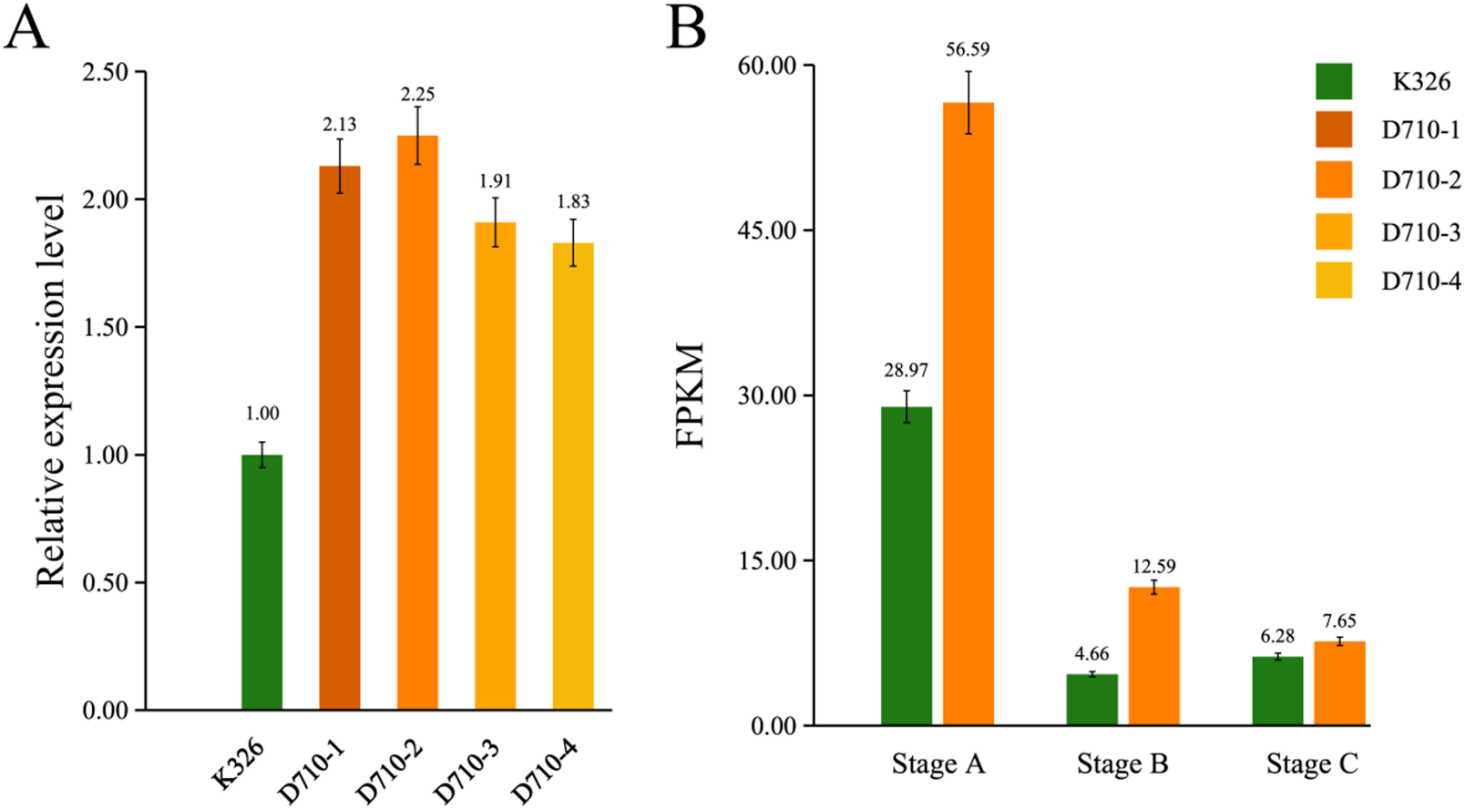
Expression analysis of *NtGLK85* in wild-type K326 and transgenic line D710. (**A**) Relative expression levels of *NtGLK85* in K326 and D710; (**B**) FPKM values of *NtGLK85* in K326 and D710 at three developmental stages. Stage A: 30 days post-transplantation, Stage B: 45 days post-transplantation, Stage C: 65 days post-transplantation. Error bars represent the means ± SD (*n* = 3); The relative expression level of *NtGLK85* in transgenic lines was calculated using K326 as the calibrator (set as 1.00), fold-changes are shown above the bars.

The analysis of qRT-PCR showed that the expression level of *NtGLK85* was significantly increased in all transgenic lines (D710-1/2/3/4). Among these, D710-2 exhibited the most pronounced upregulation of *NtGLK85* expression (Fig. 3A). To further validate the overexpression of *NtGLK85*, we quantified its expression level at three developmental stages in both D710 and K326 using RNA-Seq data. Expression levels represented as Fragments Per Kilobase of transcript per Million mapped reads (FPKM) (Fig. 3B). The results showed that transgenic line D710 had higher FPKM values than K326 at the corresponding time points (Fig. 3B), confirming that *NtGLK85* was significantly upregulated in transgenic line D710 compared to the wild-type K326.

Based on the two-year plant height statistics (Fig. 1), chlorophyll content measurements (Fig. 2), and qRT-PCR results (Fig. 3A), D710-2 (hereafter referred to as D710) was selected for further study.

### Changes in the biochemical properties of D710

**Fig. 4:**
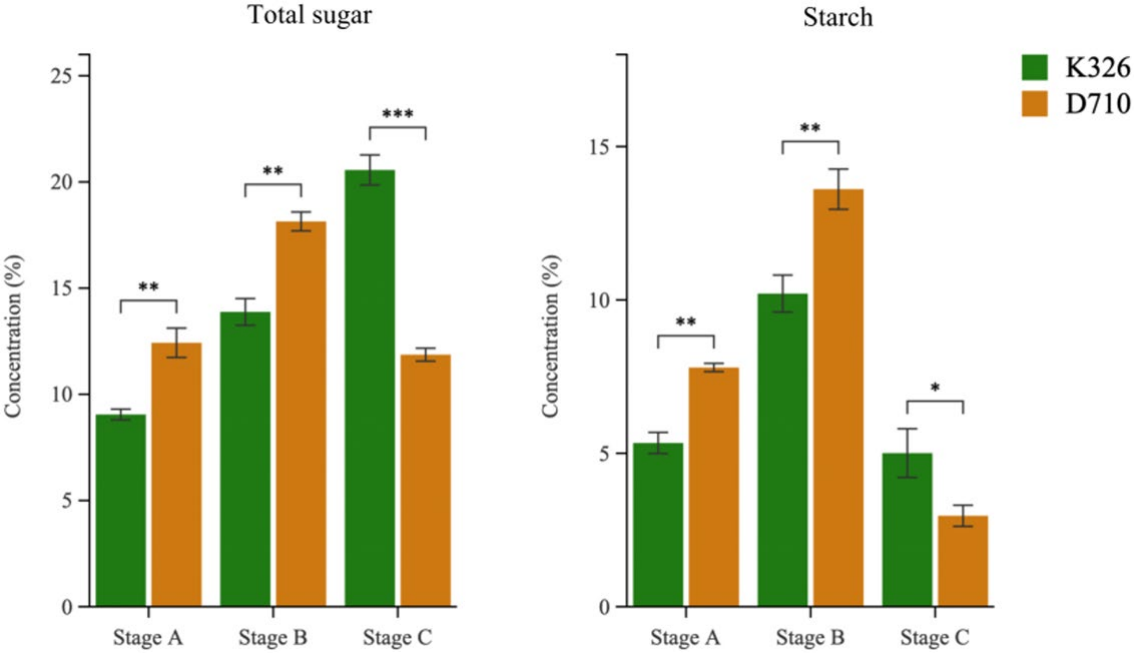
Biochemical substances of K326 and D710 at different stages. Error bars represent the means ± SD (*n* = 3); Statistical significance was assessed using Student’s t-test, with significance levels denoted as follows: * for *P* ≤ 0.05, ** for *P* ≤ 0.01, and *** for *P* ≤ 0.001.

Investigation of biochemical properties revealed that during stages A and B, the total sugar content in D710 was significantly higher than that in K326, with highly significant differences. However, at stage C, the total sugar content in D710 was lower than in K326, showing extremely significant differences. Similarly, starch content in D710 was higher than in K326 during stages A and B, with highly significant differences, but lower at stage C, with a significant difference (Fig. 4). Notably, the starch and total sugar contents in D710 showed an increasing trend during stages A and B, followed by a decline at stage C. In contrast, K326 exhibited a gradual increase in total sugar content throughout all developmental stages, while starch content increased during stages A and B, then decreased at stage C.

### Quality assessment of sequencing data

**Fig. 5:**
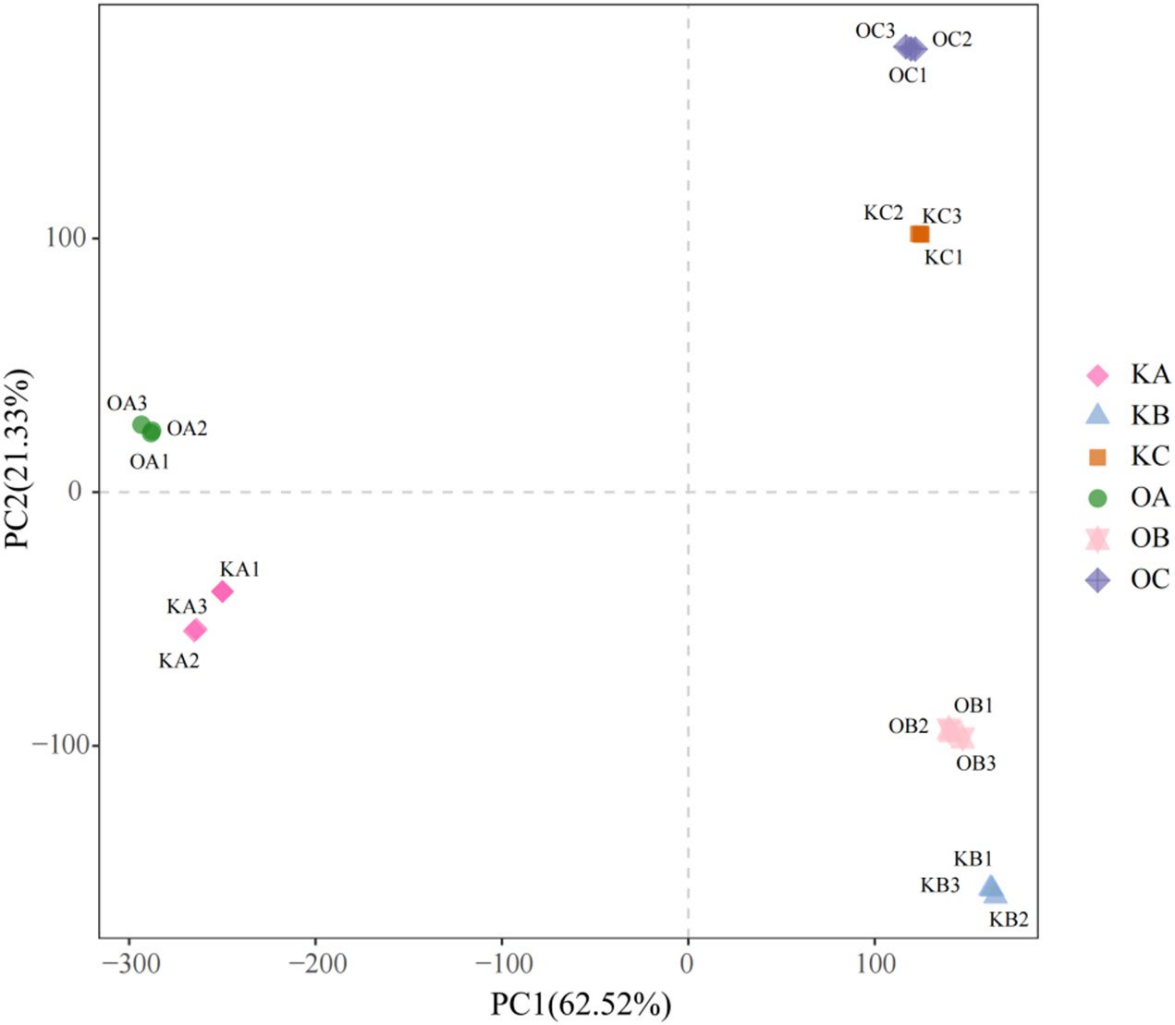
Principal component analysis (PCA). OA, OB, and OC denote the three developmental stages of the transgenetic line D710, respectively, while KA, KB, and KC represent the corresponding stages of the control K326.

A total of 380,087,228 raw read pairs were initially obtained, and 374,627,262 clean reads were passed quality control (Table 1). The clean reads had an average sequencing error rate of 0.03%, with Q20 exceeding 97%, and Q30 reaching 92.89% for OB1, and greater than 93% for all other samples. More than 93% of clean reads were successfully mapped to the reference genome with more than 89% aligning to the unique positions. Subsequently, gene expression analysis was conducted using these uniquely mapped reads. Principal component analysis (PCA) showed well-clustering of three biological replicates from the same sample and period, with some even overlapped (Fig. 5), indicating the reliability of our RNA-seq data and its suitable for further analysis.

**Table 1:** Sequencing results of samples.

Sample	Raw Reads	Clean Reads	Error Rate (%)	Q20 (%)	Q30 (%)	Total mapped (%)	Uniquely mapped (%)
KA1	41,890,510	41,329,658	0.03	97.67	93.48	94.38	89.81
KA2	39,323,920	38,672,938	0.03	97.53	93.13	95.55	90.9
KA3	44,322,568	43,706,044	0.03	97.64	93.46	95.46	90.75
KB1	41,357,100	40,801,804	0.03	97.57	93.22	93.64	89.05
KB2	41,338,654	40,676,564	0.03	97.62	93.4	94.48	89.56
KB3	44,418,570	43,768,578	0.03	97.55	93.2	94.42	89.67
KC1	41,451,626	40,815,360	0.03	97.48	92.99	95.54	90.76
KC2	42,535,542	41,923,652	0.03	97.64	93.36	95.56	91.16
KC3	44,079,530	43,442,496	0.03	97.69	93.53	95.09	90.54
OA1	43,358,094	42,853,996	0.03	97.61	93.25	93.89	89.54
OA2	42,986,698	42,427,172	0.03	97.47	93.01	93.46	89.11
OA3	39,740,252	39,259,800	0.03	97.65	93.45	94.99	90.42
OB1	40,011,374	39,349,650	0.03	97.38	92.89	95.57	91.26
OB2	41,846,502	41,311,164	0.03	97.64	93.43	95.54	91.18
OB3	42,155,242	41,441,486	0.03	97.6	93.35	95.53	89.03
OC1	43,406,704	42,778,120	0.03	97.78	93.76	95.14	90.69
OC2	43,527,198	42,874,840	0.03	97.71	93.58	94.46	90.32
OC3	42,424,372	41,821,202	0.03	97.66	93.4	95.1	90.85

Note: The three stages of D710 were named OA, OB, and OC, respectively, while the control group K326 was named KA, KB, and KC.

### Differentially expressed gene

**Fig. 6:**
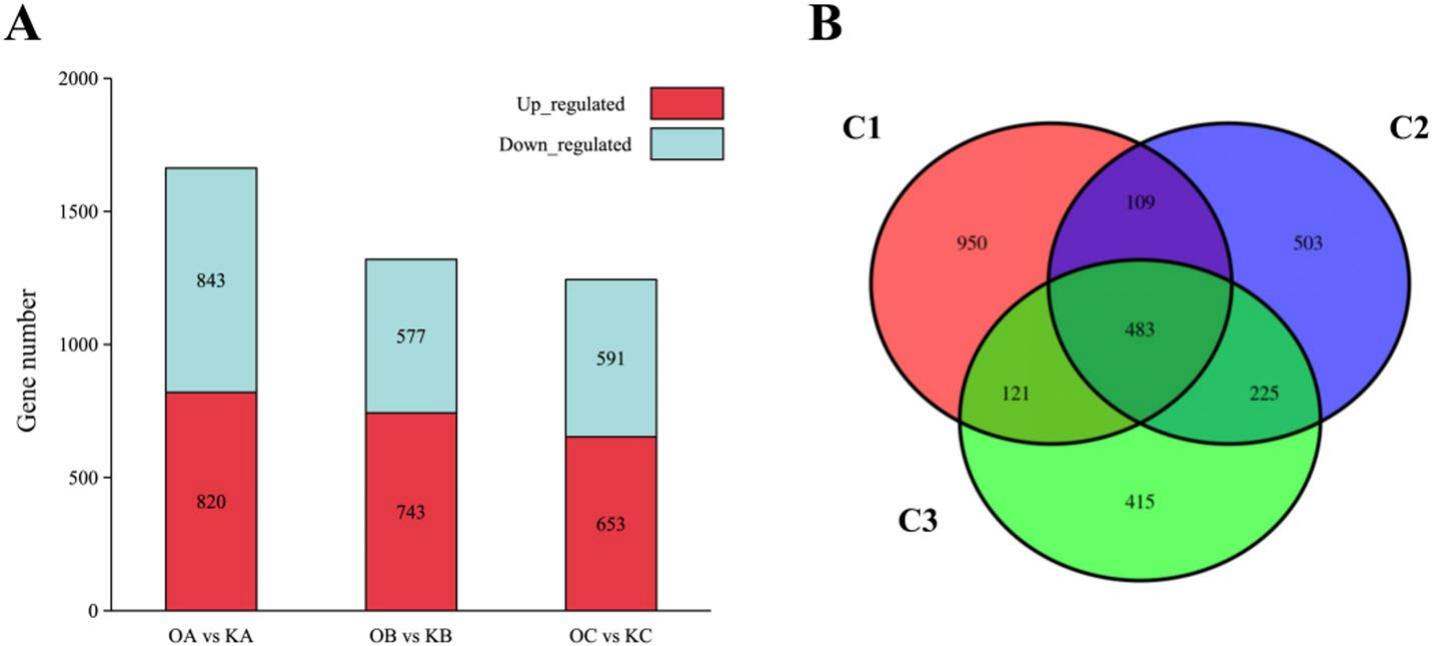
Differentially expressed genes (DEGs) in each comparison: (A) Histogram; (B) Venn diagram.

To investigate differential gene expression induced by overexpression of the *NtGLK85* gene, three pairwise comparisons were conducted, namely, C1: OA vs. KA; C2: OB vs. KB; C3: OC vs. KC. In C1, a total of 1,663 differential expressed genes (DEGs) (Table S1) were identified, including 820 up-regulated genes and 843 down-regulated genes (Fig. 6A); in C2, a total of 1,320 DEGs (Table S1) were identified, with 743 up-regulated genes and 577 down-regulated genes (Fig. 6A); in C3, a total of 1,244 DEGs (Table S1) were identified, including 653 up-regulated genes and 591 down-regulated genes (Fig. 6A). Notably, in C1, the number of up-regulated genes was less than that of down-regulated genes, whereas in C2 and C3, more genes were up-regulated upon overexpression of the *NtGLK85* gene (Fig. 6A). The Venn diagram showed 483 common DEGs across all three comparisons, while C1 had 950 unique DEGs, C2 had 503 unique DEGs, and C3 had 415 unique DEGs (Fig. 6B).

### GO enrichment analysis

Gene Ontology (GO) analysis showed that the differential expressed genes (DEGs) in C1 were significantly enriched in 22 GO terms (p-value < 0.01) (Table S2), including 10 terms related to biological processes (BP) and 12 related to molecular functions (MF). Among the BP terms, most were related to processes such as cell growth, division, response to environmental changes, and maintenance of overall physiological functions. In C2, the DEGs were significantly enriched in 40 GO terms (p-value < 0.01) (Table S2), including 26 BP terms, 3 cellular components (CC) terms, and 11 MF terms. Among the BP terms, several GO terms were related to the synthesis of genetic material, including DNA-templated transcription and elongation (GO:0006354); transcription elongation from RNA polymerase II promoter (GO:0006368); mRNA transport (GO:0051028); and mRNA export from the nucleus (GO:0006406). Additionally, several GO terms involved the transport of energy substances, such as polysaccharide transport (GO:0015774); polysaccharide localization (GO:0033037); and protein export from the nucleus (GO:0006611). Notably, in CC, DEGs were significantly enriched in the transcription elongation factor complex (GO:0008023). In MF, several GO terms were related to the enzymatic activities involved in the synthesis and degradation of genetic material, including DNA helicase activity (GO:0003678); DNA-dependent ATPase activity (GO:0008094); and helicase activity (GO:0004386). The results indicated significant differences in the transformation and synthesis of genetic material between D710 and K326 during the C2 phase. Furthermore, the regulated genetic information subsequently impacted carbohydrate transformation and synthesis, potentially explaining the phenotypic differences observed in D710. In C3, the identified DEGs were significantly enriched in 14 GO terms (p-value < 0.01) (Table S2), including 8 BP terms, 5 MF terms, and 1 CC term. In addition, the DEGs caused by NtGLK85 gene overexpression were primarily enriched in biological processes and molecular functions, with fewer associations found in cellular components. Compared to C1 and C3, C2 exhibited enrichment in a greater number of GO terms.

**Fig. 7:**
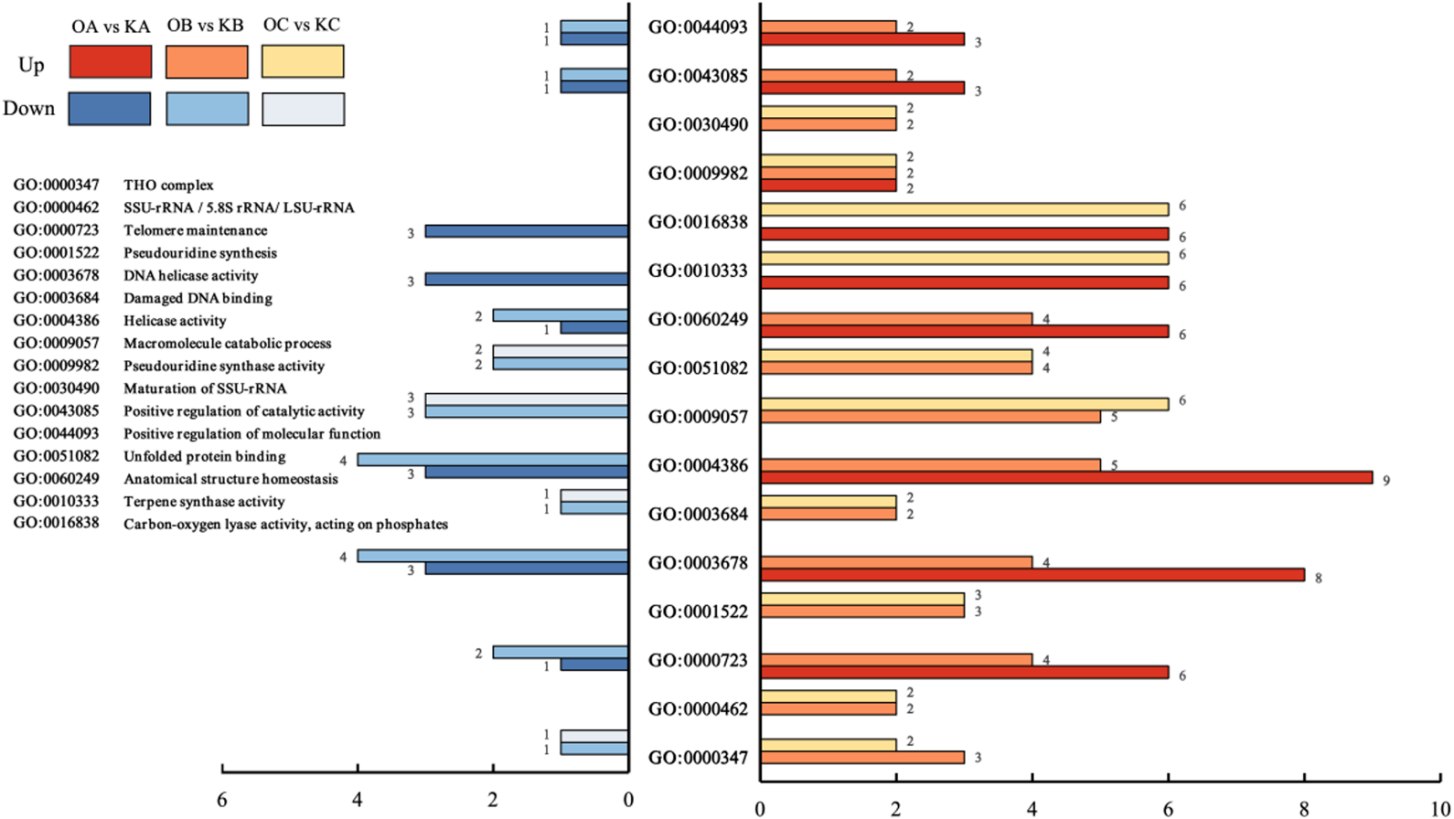
Significant GO terms common in at least two of the three pairs of comparison.

Additionally, 16 common GO terms (p-value < 0.01) shared by at least two of the three comparisons were further studied (Fig. 7).

Among them, pseudouridine synthase activity (GO:0009982) was the common GO term for C1, C2, and C3, while others were GO terms common to two of the three comparisons. There were six common GO terms between C1 and C2, four (GO:0000723;GO:0043085༛GO:0044093༛GO:0060249) were closely related to the regulation and maintenance of cellular functions, and two (GO:0003678༛GO:0004386༉ were related to DNA helicase activity. Between C1 and C3, there were two common GO terms (GO:0010333༛GO:0016838༉, each regulating the activity of different biological enzymes. Between C2 and C3, there were seven common GO terms, four (GO:0000462༛GO:0001522༛GO:0009057༛GO:0030490༉of these GO terms were associated with rRNA processing, modification, and macromolecule degradation in cells. Additionally, GO:0003684 (Damaged DNA binding) describing the specific recognition and binding of certain proteins or molecules to damaged DNA regions, while GO:0051082 (Unfolded protein binding) describing the recognition and binding of molecular chaperones or other related proteins to improperly folded or incompletely folded proteins; and one GO term (GO:0000347༉was related to the THO complex (Fig. 7).

Overall, the number of up-regulated DEGs exceeded the number of down-regulated DEGs. For example, in GO:0000347 (THO complex), 4 DEGs were enriched in C2, with 3 up-regulated and 1 down-regulated. In C3, 2 up-regulated DEGs and 1 down-regulated DEG were enriched in this GO term.

### KEGG pathway enrichment and related DEGs analysis

The KEGG pathway enrichment analysis showed that 11 pathways were enriched in C1, including the cutin suberine and wax biosynthesis, photosynthesis, signaling, terpenoid biosynthesis and metabolism, biosynthesis of secondary metabolites, and flavonoid biosynthesis (Figure S1). In C2, DEGs were enriched in 9 pathways, 5 of which overlapped with C1, including 3 related to the terpenoid biosynthesis and metabolism. C3 exhibited enrichment in 5 pathways with DEGs (Figure S1), including riboflavin metabolism, phenylpropanoid biosynthesis, diterpenoid biosynthesis, monoterpenoid biosynthesis, and biosynthesis of secondary metabolites. To further investigate the function regulated by NtGLK85 in plant growth and development, five KEGG pathways related to plant growth and development were selected from the KEGG pathways enriched with differentially expressed genes (DEGs) for further study (Table S3). These pathways include photosynthesis (map00195), fatty acid biosynthesis (map00061), cutin, suberine, and wax biosynthesis (map00073), carbon fixation in photosynthetic organisms (map00710), and fatty acid elongation (map00062)^36,37^.

**Fig. 8:**
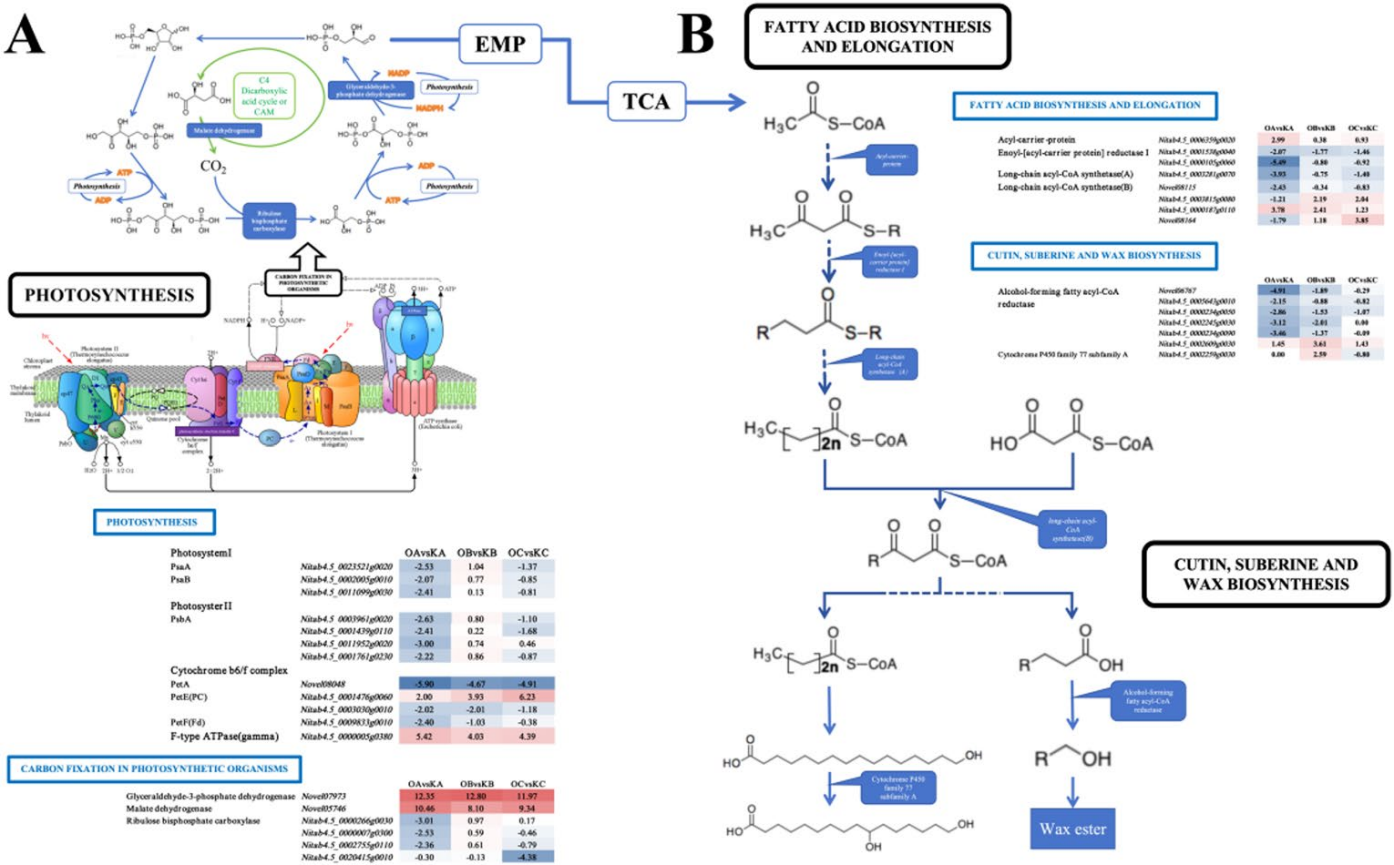
KEGG pathway correlation diagram. OA, OB, and OC denote the three developmental stages of the transgenetic line D710, respectively, while KA, KB, and KC represent the corresponding stages of the control K326.

In the photosynthesis pathway (Fig. 8A), due to the overexpression of *NtGLK85*, the expression of 7 genes related to photosystems I and II was down-regulated in the C1 comparison. In the cytochrome b6/f complex, *Novel08048*, which is involved in PetA synthesis, was down-regulated in all three comparisons, while *Nitab4.5_0001476g0060*, associated with PetE (PC) synthesis, was up-regulated in all comparisons. Additionally, *Nitab4.5_0000005g0380*, which affects ATPase activity, was significantly up-regulated in all comparisons.

In the carbon fixation in photosynthetic organism pathway (map00710)^36,37^, the expression levels of *Novel05746*, which influences malate dehydrogenase activity, and *Novel07973*, which affects the composition and activity of glyceraldehyde-3-phosphate dehydrogenase, were up-regulated in all comparisons. Conversely, four genes related to ribulose bisphosphate carboxylase were down-regulated, including *Nitab4.5_0000266g0030*, *Nitab4.5_0000007g0300*, and *Nitab4.5_0002755g0110* in the C1 comparison, and *Nitab4.5_0020415g0010* in the C3 comparison.

In the fatty acid biosynthesis (map00061)^36^ and fatty acid elongation (map00062)^37^ pathways (Fig. 8B), *Nitab4.5_0006359g0020*, related to the acyl carrier protein, was up-regulated in the C1 comparison. In contrast, *Nitab4.5_0001538g0040* and *Nitab4.5_0000105g0060*, which encode enoyl-[acyl-carrier protein] reductase I, were down-regulated, along with *Nitab4.5_0003281g0070*, which affects long-chain acyl-CoA synthetase (A). Additionally, Novel08115, related to long-chain acyl-CoA synthetase (B), was also down-regulated. *Nitab4.5_0003815g0080* was up-regulated in both C2 and C3, while *Nitab4.5_0000187g0110* was up-regulated in C1 and C2, and *Novel08164* was down-regulated in C1 and up-regulated in C3.

In the cutin, suberine, and wax biosynthesis pathway (map00073) (Fig. 8B)^37^, DEGs related to alcohol-forming fatty acyl-CoA reductase were down-regulated in C1, except for *Nitab4.5_0002609g0030*, which was up-regulated in all comparisons. Additionally, *Nitab4.5_0002259g0030*, which influences cytochrome P450 family 77 subfamily A, was up-regulated in C2. Table S4 lists the expression levels and functional annotation information of these important DEGs related to the five selected pathways. These DEGs may play crucial roles in plant growth and development.

### Quantitative real-time PCR analysis of selected DEGs

**Fig. 9:**
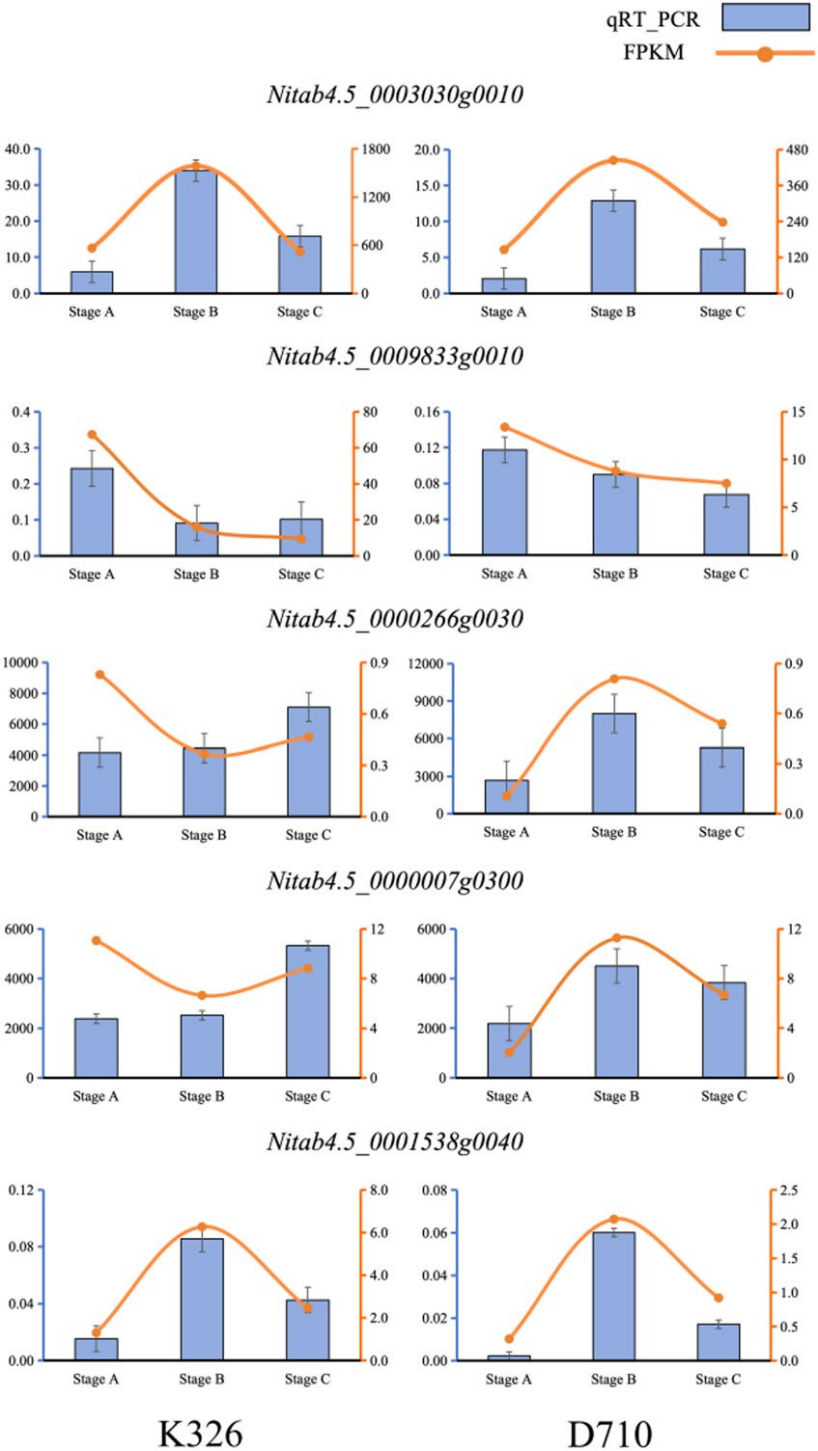
qPCR and FPKM values of five selected differentially expressed genes at three development stages. Stage A: 30 days post-transplantation, Stage B: 45 days post-transplantation, Stage C: 65 days post-transplantation.

To validate the reliability of the transcriptome data, we designed primers for 5 differentially expressed genes (Table S5) and conducted quantitative real-time PCR analysis. We found that the expression trends of these genes at stages A, B, and C were consistent with the changes in their FPKM values observed in the transcriptome data (Fig. 9). This result further confirmed the reliability of our transcriptome data and supports the conclusions drawn from our transcriptome data analysis.

## Discussion

Previous studies have demonstrated the key role of *GLK* in chloroplast development^21–23^. Photosynthesis is the primary process through which plants acquire energy; converting light energy into chemical energy stored within their cells for various life activities. Overexpression of the *GLK* gene leads directly to chlorophyll accumulation in non-photosynthetic tissues^21^, thereby influencing plant photosynthetic efficiency. In this study, we observed that overexpression of *NtGLK85* significantly increased chlorophyll content and promoted sugar accumulation, ultimately accelerating the growth and development rates of transgenic plants. Although variations in plant height were observed between the two years, likely due to differences in temperature and humidity, the transgenic strains consistently exhibited significant growth advantages over the control group in both 2022 and 2023. Transcriptome analysis further revealed several differentially expressed genes (DEGs) related to ATPase activity in stages C1 and C3. For instance, Nitab4.5_0000005g0380 was found to influence the ATP synthesis rate during photosynthesis, directly impacting subsequent photosynthetic carbon assimilation rates. These findings underscore the critical role of the *NtGLK85* gene in the regulatory network of photosynthesis, highlighting its potential to modulate key metabolic processes in plants.

**Fig. 10:**
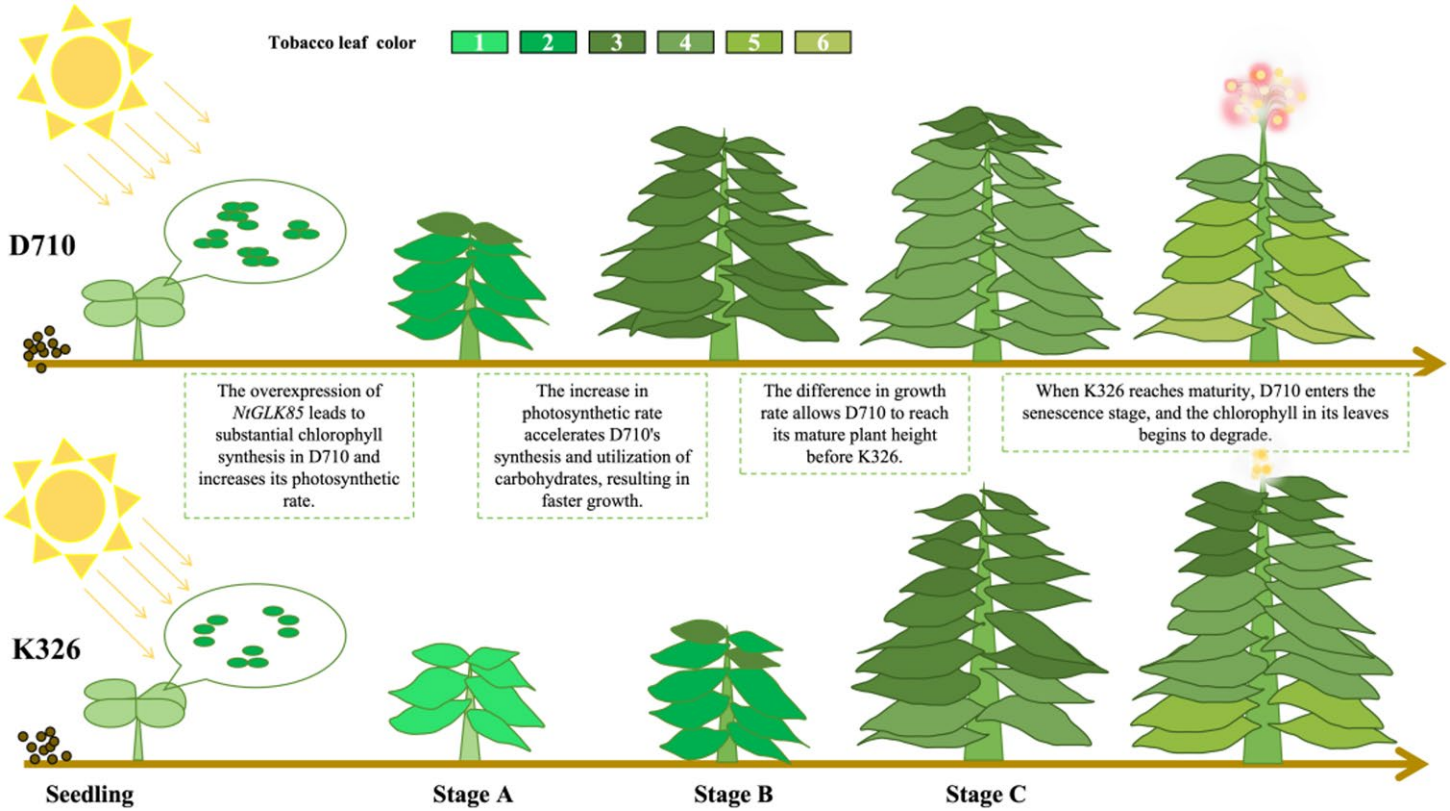
Growth diagram of D710 and K326.

Carbon fixation, a critical metabolic process in photosynthesis, converts light energy into chemical energy, generating ATP and NADPH. These molecules serve as essential energy carriers and reducing agents, driving the synthesis of carbohydrates—key energy sources for various plant life activities^38^. In this study, during stages A and B, the starch content utilized for energy storage was higher in D710 compared to K326. However, as stage C progressed, the starch level in both materials decreased, with D710 exhibiting a lower starch content than K326. Similarly, although the total sugar content in K326 steadily increased over time, it remained beneath that of D710 during stages A and B. By stage C, the total sugar content in D710 experienced a notable decrease, falling below that of K326 at this stage. Furthermore, we observed that D710 developed flower buds at this stage. This suggested that while K326 continued to synthesize carbohydrate substances and began consuming starch at stage C, whereas D710 had already utilized a substantial portion of its starch and other carbohydrates, advancing into the reproductive phase. These findings highlight distinct metabolic strategies between the two materials, with D710 exhibiting earlier carbohydrate utilization and reproductive development compared to K326. (Fig. 10). Notably, our transcriptome data revealed that the KEGG analysis of DEGs in C2 was enriched in carbohydrate transport terms, with several DEGs also showing enrichment in the carbon fixation pathway (Fig. 8). For example, the expression levels of Novel07973 significantly increased at all three stages, and this gene is closely related to glyceraldehyde-3-phosphate dehydrogenase, which catalyzes the dehydrogenation of glyceraldehyde-3-phosphate in the Calvin cycle, producing NADPH and regulating the synthesis rate of acetyl-CoA. The results indicate that the overexpression of the *NtGLK85* gene not only affects photosynthesis but also regulates the synthesis and transport of carbohydrate substances.

Fatty acids are one of the main sources of energy in plants. Photosynthesis produces glucose, which is broken down into pyruvate through the glycolysis pathway and then synthesized by acetyl-CoA^39^. In the initial stages, D710 exhibited an accumulation of photosynthetic compounds, which in turn elevated the photosynthetic rate, leading to an acceleration in the synthesis and conversion of carbohydrate substances. By 65 days post-transplantation, the level of starch and total sugar in D710 had decreased relative to K326, likely due to the decomposition and utilization of carbohydrate substances for the synthesis of fatty acids and other biomolecules. This observation is supported by our transcriptome data, which revealed significant enrichment of differentially expressed genes (DEGs) in the fatty acid elongation and fatty acid biosynthesis pathways during C1 and C2 comparisons. Among these DEGs, *Nitab4.5_0006359g0020* is related to the acyltransferase domain and belongs to acyl carrier proteins. *Nitab4.5_0001538g0040* and *Nitab4.5_0000105G0060* are functionally annotated as glucose dehydrogenase and enoyl reductase I. Additionally, other DEGs are related to long-chain acyl-CoA synthetase. The long-chain fatty acids synthesized through these pathways subsequently participate in the biosynthesis of cutin, suberin, and wax, which contribute to the synthesis of cellulose and lignin. The substantial consumption of carbohydrates also accelerates the synthesis of long-chain fatty acids, which serve as precursors for lignin synthesis, thereby influencing lignin transformation and accumulation. Studies have demonstrated that lignin plays a critical role in plant height, as evidenced by lignin-deficient mutants exhibiting dwarfism, infertility, xylem collapse, and an inability to stand upright^40^. Agronomic trait analysis revealed that D710 reached maturity in height earlier than K326, suggesting potential regulation of lignin synthesis, which is closely associated with plant height growth. Furthermore, we identified DEGs related to this phenomenon in the cutin, suberin, and wax biosynthesis pathways, confirming the impact of *NtGLK85* on lignin synthesis. These findings highlight the multifaceted role of *NtGLK85* in regulating carbohydrate metabolism, fatty acid biosynthesis, and lignin synthesis, ultimately influencing plant growth and development.

Previous study have indicated that the expression level of gene can be attributed to several potential factors, including post-transcriptional regulation, epigenetic modifications, tissue-specific modifications, or developmental stage-specific influences on gene expression^41^. For instance, mechanisms such as mRNA stability, degradation, or translational efficiency may vary across different developmental stages^42^, even under the control of a constitutive promoter. Supporting this, our previous research demonstrated distinct expression patterns of *NtGLK85* in tobacco leaves across five senescence stages^35^, highlighting the complexity of gene regulation during development. In this study, the expression of *NtGLK85* in the K326 control also exhibited natural variation across developmental stages, suggesting that *NtGLK85* expression is intrinsically linked to plant growth and development. Although the constitutive promoter ensures continuous transcriptional activity, the FPKM values of *NtGLK85* still varied across the three developmental stages in the overexpression line. Importantly, these values remained consistently higher in the transgenic line D710 compared to the wild-type K326 at all time points, indicating the stability of the transgenic line.

It is noteworthy that the overexpression of *NtGLK85* in transgenic lines resulted in a relatively moderate increase in expression levels (~ 2.4-fold). Despite this modest elevation, significant and widespread physiological changes were observed, including the palnt height and the content of chlorophyll. This phenomenon can likely be attributed to the functional role of *NtGLK85* as a member of the GARP superfamily of nuclear transcription factors^11^. Previous studies have established that GLK genes play a critical role in chloroplast development in angiosperms^21–23^, underscoring their importance in plant physiology. Our previous further supports this observation, demonstrating that *NtGLK85* exhibits high expression levels in leaves, with its expression closely linked to the degree of leaf senescence^35^. This suggests that even a relatively small increase in expression (e.g., two-fold) can have profound physiological implications, potentially due to the gene’s regulatory influence on key developmental and metabolic processes.

## Materials and methods

### Vector construction and tobacco transformation

To construct the *NtGLK85* (*Nitab4.5_0010689g0010*) overexpression vector (abbreviated as D710), the full-length cDNA of *NtGLK85* was cloned from the cDNA of the tobacco variety K326 using GLK85-F/GLK85-R primers (Table S6) via PCR. The amplified product was digested and cloned into the [pBWA(V)HS-GLK85] vector to construct an overexpression vector driven by the 35 S promoter. Transgenic tobacco plants were generated using the Agrobacterium-mediated leaf disc method^43^. Transgenic lines were identified through hygromycin resistance screening and validated by qRT-PCR. The 2^−∆∆CT^ method was used to calculate relative expression levels^44^, with actin serving as the internal reference gene. The *NtGLK85* primers used for qRT-PCR are shown in Supplementary Table S7. As a result, four independent transgenic lines, named D710-1 to D710-4, were successfully obtained.

### Planting and morphological phenotype investigation of Transgenic lines

Seeds from the T2 generation of the four transgenic lines were sown in the laboratory and cultivated using conventional methods. Following a 60-day seedling stage, 30 plants from each transgenic line (D710-1/2/3/4) and the wild-type K326 were selected and transplanted into the field for continuous cultivation during the 2022 and 2023 growing seasons. Two field experiments were performed in the experimental farm of Lixin Town, Jianning County, Sanming City, Fujian Province (E116.843803, N26.846884) in 2022 and 2023. In 2022, the plants were grown under the following conditions: average temperature range of 22–28 °C, relative humidity of 65–75%, light intensity of 1200–1500 µmol/m^2^/s, and soil moisture maintained at 60–70% of field capacity. The plants were grown using a standard row planting method with 50 cm between plants and 120 cm between rows. In 2023, slightly different conditions were used: average temperature range of 20–26 °C, relative humidity of 70–80%, light intensity of 1100–1400 µmol/m^2^/s, and soil moisture maintained at 65–75% of field capacity. The planting method remained consistent with the 2022 trial. Under the same standard field management practices, plant height of all transgenic lines and wild-type plants was investigated and measured at 30 days (Stage A), 45 days (Stage B), and 65 days (Stage C) after transplantation.

### Sampling and physiological indexes analysis

Based on the morphological difference analysis between the *NtGLK85* overexpression materials and the control, samples of D710-1/2/3/4 and K326 were collected at 30 days (Stage A), 45 days (Stage B), and 65 days (Stage C) after transplantation. Each biological sample consisted of 5 plants, with a total of 3 replicates. The three stages of D710 were named OA, OB, and OC, respectively, while the control group K326 was named KA, KB, and KC (Table S8). RNA sequencing was performed on these samples. New leaves at Stage A were selected from both D710 and K326 for sectioning. Imaging of the leaf sections was carried out at room temperature using a Leica SP8 X inverted confocal microscope equipped with an argon laser (Leica, Wetzlar, Germany). Chlorophyll fluorescence was excited with a 552 nm laser line and emission was captured within the 650–680 nm range. Images were digitally captured and processed using Leica Application Suite Advanced Fluorescence Lite software (Leica Microsystems). The fluorescence intensity was quantified using ImageJ software (http://rsbweb.nih.gov/ij/)^45^. Finally, the physiological indexes of chlorophyll, total soluble sugar, and starch in D710 and K326 were measured across the three stages. Chlorophyll was extracted by soaking the leaves in 95% ethanol. The supernatant was collected and absorbance was measured at wavelengths of 649 nm and 665 nm using a spectrophotometer (UV-1780). Chlorophyll content was calculated and averaged across three biological replicates. Total sugar and starch content were measured according to the method outlined in the Chinese tobacco industry standard (YC/T159-2002).

### RNA sequencing and data analysis

RNA was extracted using the TRIzol method (Invitrogen, CA, USA) and treated with DNase I (Takara, Kusatsu, Japan) to remove DNA. A total of 18 samples was extracted (Table S8). RNA quality and integrity were assessed using a NanoDrop spectrophotometer (Thermo Scientific, DE, USA). The NEBNext^®^ Ultra RNA Library Prep Kit (Illumina^®^) was used for library preparation following the manufacturer’s instructions. Samples were sequenced on Illumina HiSeq 2000 performed by Biomarker Technologies (https://www.biomarker.com.cn/, BioMarker, Beijing, China). Clean readings are mapped to the tobacco reference genome (Edwards 2017) through STAR^46^ (https://solgenomics.net/organism/Nicotiana_Attenuata/home)^47^. For gene expression analysis, the numbers of matched reads were normalized by the fragments per thousand base transcripts per million mapped fragments (FPKM). Gene ontology (GO) analysis was performed based on the Gene Ontology Database (https://www.geneontology.org/). The GO seq^48^ was used to perform GO enrichment analysis on DEGs based on the Wallenius non central hypergeometric distribution of the GO seq R package. The KEGG metabolic pathways associated with DEGs obtained from different samples were analyzed (http://www.kegg.jp/kegg/pathw ay.html)^49^. KOBAS^50^ software was used to test the statistical enrichment of differentially expressed genes in the KEGG pathway.

### Quantitative Real-Time PCR analysis

To validate the quality of RNA-seq data and the expression levels of candidate genes (Table S5), qRT-PCR was performed. cDNA synthesis was carried out using a PrimeScript™ RT reagent kit with gDNA eraser (Takara, Japan) following the manufacturer’s instructions. The cDNA samples were then assayed by qRT-PCR using SYBR Premix Ex Taq (Takara) on the ABI 7500 Fast Real-Time PCR System (Applied Biosystems, USA). Three biological replicates and three technical replicates were tested. The relative expression level of the detected gene was calculated using the 2^−ΔΔCt^ method^51^.

### Statistical analysis

Differences between groups were assessed for statistical significance using Student’s t-test with significance levels defined as *P* ≤ 0.05. Unsupervised principal component analysis (PCA) was performed using the statistics function within R (www.r-project.org; version 3.5.1)^52^. The data were unit variance scaled before performing unsupervised PCA. Differentially expressed genes (DEGs) were identified using the criteria of |log2(fold change)|≥2 and false discovery rate (FDR) ≤ 0.01.

## Conclusions

The overexpression of the *NtGLK85* gene in the K326 tobacco variety results in significant improvements in plant growth and development in the different stages of tobacco development. The D710 transgenic line, which overexpresses *NtGLK85*, showed a faster growth rate and a shorter growth cycle compared to the wild-type K326. This enhancement is associated with increased chlorophyll content, higher levels of total sugars, and starch during the early growth stages. Although these levels decreased in the later stages, the overall plant height remained remarkable. RNA-Seq analysis identified numerous DEGs and key pathways affected by *NtGLK85* overexpression, including those involved in photosynthesis and carbohydrate metabolism. The results underscore the crucial role of *NtGLK85* in enhancing carbohydrate accumulation, leading to accelerated plant growth and development. This research highlights the potential of *NtGLK85* as a target for improving crop growth and yield through genetic manipulation.

## Supplementary Information

Below is the link to the electronic supplementary material.

Supplementary Material 1

Supplementary Material 2

Supplementary Material 3

Supplementary Material 4

Supplementary Material 5

Supplementary Material 6

Supplementary Material 7

Supplementary Material 8

Supplementary Material 9

Supplementary Material 10

## Data Availability

The sequence data supporting the findings of this study have been deposited in the NCBI Sequence Read Archive (SRA) with the primary accession code PRJNA1147468.

## Abbreviations

GLK: Golden 2-like
GARP: GOLDEN2, ARR-B, Psr1
SlGLK: GLK genes of Solanum lycopersicum
AtGLK: GLK genes of Arabidopsis
ZmGLK: GLK genes of Zea mays L
NtGLK: GLK genes of Nicotiana tabacum
D710: The material with overexpression of the NtGLK85 gene
K326: The control material with wild-type
DEGs: The differential expressed genes
GO: Gene Ontology
BP: Biological Processes
MF: Molecular Functions
CC: Cellular Components
KEGG: Kyoto Encyclopedia of Genes and Genomes
FPKM: Fragments Per Kilobase of transcript sequence per Millions base pairs sequenced
qRT-PCR: Quantitative real-time PCR
