# Supplementary material for: Analysis of mechanisms underlying accelerated plant growth induced by NtGLK85 overexpression in tobacco
Source: Sci Rep. 2025 Nov 18;15:40584. doi: 10.1038/s41598-025-24323-x (PMC12627591; doi:10.1038/s41598-025-24323-x)
Supplement: Supplementary file 9 — Supplementary Material 9 [file 41598_2025_24323_MOESM9_ESM.docx]

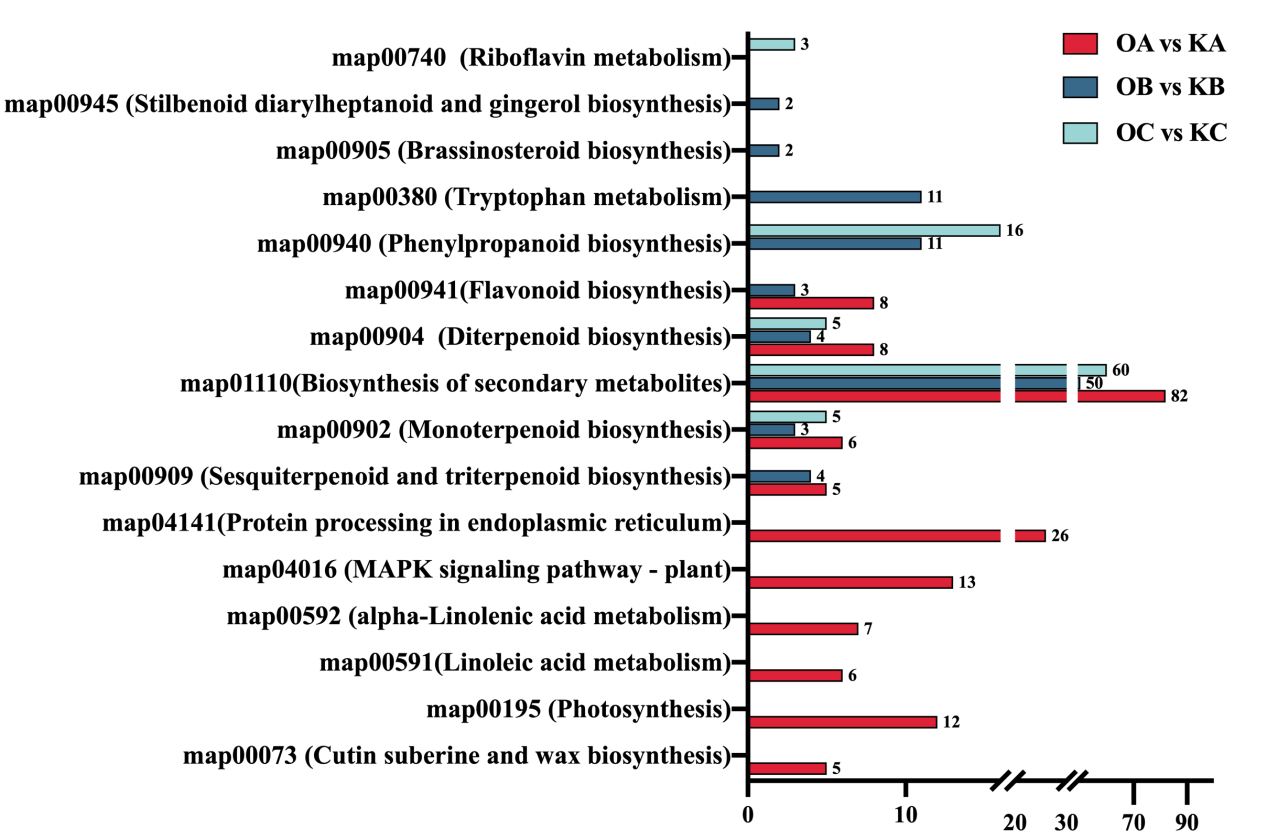
**Figure S1.** KEGG pathway enrichment in the three pairwise comparisons.

OA, OB, and OC denote the three developmental stages of the transgenic line D710, respectively, while KA, KB, and KC represent the corresponding stages of the control K326.
