# Supplementary figures and images for: Analysis of mechanisms underlying accelerated plant growth induced by NtGLK85 overexpression in tobacco
Source: Sci Rep. 2025 Nov 18;15:40584. doi: 10.1038/s41598-025-24323-x (PMC12627591; doi:10.1038/s41598-025-24323-x)

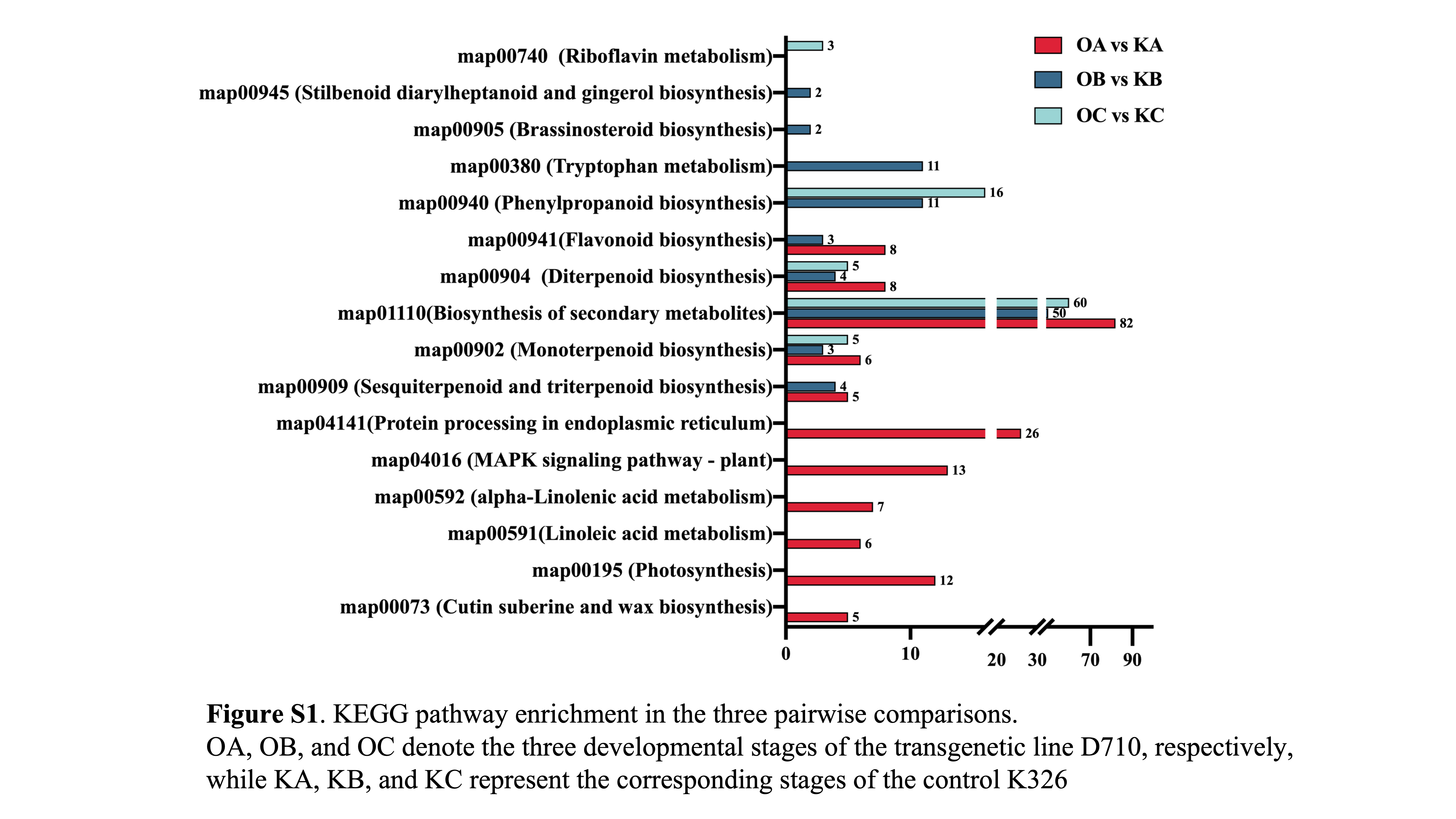

Supplement: Supplementary file 10 — Supplementary Material 10 [file 41598_2025_24323_MOESM10_ESM.zip › Supplementary File_σë»μ£1⁄4/Figure S1.png]
